# Draft Genome Sequences of New Genomospecies “*Candidatus* Pectobacterium maceratum” Strains, Which Cause Soft Rot in Plants

**DOI:** 10.1128/genomeA.00260-18

**Published:** 2018-04-12

**Authors:** Fedor V. Shirshikov, Aleksei A. Korzhenkov, Kirill K. Miroshnikov, Anastasia P. Kabanova, Alla P. Barannik, Alexander N. Ignatov, Konstantin A. Miroshnikov

**Affiliations:** a Shemyakin & Ovchinnikov Institute of Bioorganic Chemistry RAS, Moscow, Russia; b PhytoEngineering R&D Center LLC, Rogachevo, Moscow Region, Russia; c Immanuel Kant Baltic Federal University, Kaliningrad, Russia; d Winogradsky Institute of Microbiology, Research Center of Biotechnology RAS, Moscow, Russia

## Abstract

Investigation of collections of phytopathogenic bacteria has revealed some strains distinct from known Pectobacterium spp. We report here the draft genome sequences of five such strains, isolated during the period of 1947 to 2012. Based on comparative genomics, we propose a new candidate genomospecies of the genus Pectobacterium, “*Candidatus* Pectobacterium maceratum.”

## GENOME ANNOUNCEMENT

The genus Pectobacterium is a genetically diverse group of pectinolytic phytopathogens (1) with a broad range of plant hosts (2). Within a collection of 200 bacterial isolates, we have found several strains closely related to Pectobacterium carotovorum, which were repeatedly isolated from macerated plant tissue of cabbage and potato tubers in the Moscow Region, Russia. These five strains caused large outbreaks of soft rot in fields and storage during the period of 1947 to 2012 (Table 1).

**TABLE 1:** Statistics for the draft Pectobacterium genome sequences

Strain	Origin	Yr	Size (bp)	Coverage (×)	No. of contigs	No. of CDSs^a^	GenBank accession no.
F018	Cabbage	1947	4,911,793	58	27	4,311	PDVV00000000
F131	Potato	1993	4,786,484	50	87	4,173	PDVW00000000
F135	Potato	2012	4,891,351	53	75	4,316	PDVX00000000
PB69	Potato	2012	4,993,011	44	21	4,382	PDVY00000000
PB70	Potato	2012	4,992,983	44	28	4,388	PDVZ00000000

a CDS, coding sequence.

Bacterial strains were cultivated overnight in liquid LB medium at 27°C. Genomic DNA extraction was performed using the phenol-chloroform method. The NEBNext Ultra DNA library prep kit for Illumina (New England BioLabs, Ipswich, MA) was used for DNA library construction. DNA samples were sequenced to generate 100-bp paired-end reads using the Illumina MiSeq platform. Paired-end reads were filtered and trimmed using CLC Genomics Workbench software (Qiagen, Aarhus, Denmark). The genome sequences were assembled using SPAdes software (3) and annotated using the Prokka annotation pipeline (4).

Recently, the general guidelines for using genome data in prokaryotic taxonomy were published (5). We applied these guidelines to determine the systematic positions of our strains. Pairwise calculations of average nucleotide identity (ANI) (6) and digital DNA-DNA hybridization (dDDH) using formula 2 for incompletely sequenced genomes (7) were performed. We used a set of 50 genome sequences, including those of our five strains and representative strains from all currently proposed taxa of the genera Pectobacterium and Dickeya. Based on the ANI distance dendrogram, our strains form a new, distinct clade that shares a common ancestor with P. carotovorum subsp. odoriferum. To distinguish members of the new clade from other subspecies of P. carotovorum, we calculated corresponding maximal ANI and dDDH values, as follows: P. carotovorum subsp. actinidiae, 93% and 50%, respectively; P. carotovorum subsp. brasiliense, 92% and 50%; P. carotovorum subsp. carotovorum, 95% and 65%; and P. carotovorum subsp. odoriferum, 95% and 62%. The ANI and dDDH values in comparison with those of the new species Pectobacterium polaris (8), which is closely related to P. carotovorum subsp. brasiliense, are 94% and 55%, respectively. The minimal intragroup ANI and dDDH values for our strains are 98% and 82%, respectively. Thresholds of species delineation by ANI and dDDH are 95 to 96% and 70%, respectively (5); the cutoff to distinguish subspecies by dDDH is 79% (9).

Thus, we propose a new candidate genomospecies for the new strains, “Candidatus Pectobacterium maceratum.” Based on high similarity with our strains (minimal ANI and dDDH values of 98% and 83%, respectively), the Finnish Pectobacterium strain SCC1 (10) also belongs to “*Ca.* Pectobacterium maceratum.” Considering that SCC1 is a model plant pathogen with a complete genome sequence, we propose it as a type strain of the candidate genomospecies. An interesting genome feature of all these strains is the presence of an *evf* gene, which encodes a virulence factor for persistence in the gut of Drosophila (11, 12). Perhaps, acquisition of the gene by some P. carotovorum subsp. odoriferum cells caused the divergence of “*Ca.* Pectobacterium maceratum” and evolution of the associated symbiosis with flies.

Further formal description of “*Ca.* Pectobacterium maceratum” is currently in progress.

### Accession number(s).

This whole-genome sequencing project has been deposited at DDBJ/ENA/GenBank under the accession numbers listed in Table 1. The BioProject accession numbers are PRJNA414969 (strain F018) and PRJNA415106 (strains F131, F135, PB69, and PB70).
